# Historical Circulation and Forgotten Evidence of Oropouche Virus in Colombia: Not as New as it Seems

**DOI:** 10.4269/ajtmh.25-0423a

**Published:** 2025-09-11

**Authors:** Carlos Ramiro Silva-Ramos, Álvaro A. Faccini-Martínez

**Affiliations:** Grupo de Enfermedades Infecciosas, Departamento de Microbiología, Facultad de CienciasPontificia Universidad Javeriana Bogotá, D.C., Colombia, Department of Pathology University of Texas Medical Branch Galveston, Texas E-mail: cramiro-silva@javeriana.edu.co; Servicio de Infectología Hospital Militar CentralBogotá, D. C., Colombia, Facultad de MedicinaUniversidad Militar Nueva GranadaBogotá, D.C., ColombiaE-mail: afaccini@gmail.com

Dear Editor,

We have read with great interest the manuscript by Walsh et al., “Investigating Oropouche as a Possible Etiology for Febrile Illness in a Clinical Cohort from Colombia, 2014–2015,” published in *The American Journal of Tropical Medicine and Hygiene*.^1^ In this study, a screening of serum samples from dengue infection-suspected patients and healthy controls from three municipalities (Cúcuta, Los Patios, and Ocaña) of Norte de Santander department (northeastern Colombia) was conducted, aiming to determine whether Oropouche virus (*Orthobunyavirus oropoucheense* [OROV]) circulated in Colombia prior to its first reported detection in 2017.^1^^,^^2^ Their molecular screening revealed acute OROV infection in three individuals from Ocaña municipality, suggesting earlier circulation than previously established.^1^

While the first laboratory-confirmed clinical case of OROV in Colombia was reported in 2021, from a patient sampled in 2017 in Turbaco, Bolívar,^2^ serological evidence of OROV circulation in Colombia dates back several decades. In 1961, OROV-neutralizing antibodies were detected in 27.3% (6/22) of primates sampled in Lizama municipality, Santander department, through a virus neutralization test.^3^ Additionally, several years later, between 2000 and 2004, sentinel surveillance studies revealed OROV seropositivity in 7.4% (4/54) of febrile patients in Guaviare department, and 7.3% (7/96) in Cundinamarca department, based on hemagglutination inhibition assays.^4^^,^^5^ These studies demonstrated that OROV had been circulating in Colombia for a long time.

However, despite these historical findings, OROV remains a neglected cause of acute undifferentiated febrile illness in Colombia, partly because it is not included in the list of mandatory national notifiable diseases.^6^ A large outbreak reported in Brazil between 2023 and 2024 prompted the Pan American Health Organization to recommend molecular testing for OROV in 10–30% of dengue-negative acute samples in Brazil and surrounding regions in February, 2024.^7^^,^^8^ As of February, 2025, 74 OROV cases have been reported in Colombia, mainly from Amazonas department, all of them reported during the first 22 epidemiological weeks of 2024.^9^

Interestingly, recent studies among Colombian entomological collections support the wide distribution of biting midges (*Culicoides* spp.) in the country.^10^ Among over 7,500 medically relevant insect specimens collected across Colombia, 801 belonged to the Ceratopogonidae family, with more than 90% identified as *Culicoides* spp., including *Culicoides paraensis*, the recognized OROV vector, which has been identified in eight Colombian departments: Boyacá, Caldas, Cesar, Guaviare, Magdalena, Quindío, Santander, and Tolima.^10^^,^^11^

The emergence and under recognition of OROV highlight the multifaceted challenges of arboviral surveillance in the 21st century. These findings emphasize the urgent need not only to strengthen surveillance, but also to improve diagnostic capacity, apply genomic epidemiology, and implement evidence-based clinical protocols.^12^ Ultimately, sustained investment, political commitment, and international collaboration will be essential to close these critical gaps for global health security.^12^

We commend Walsh et al. for their valuable contribution to OROV surveillance in Colombia.^1^ However, we encourage the consideration of existing historical data, which show that OROV circulation in Colombia likely pre-dated 2014 and may have been more widespread than currently recognized. These findings highlight the need for further studies to solve existing challenges on OROV emergence in Colombia, and eventually, to add scientific evidence on severe infections, neurological complications, and fetal abnormalities related to this virus.^13^
